# GATM alleviates sepsis-induced acute kidney injury via PDK4-mediated glycolytic reprogramming in renal tubular epithelial cells

**DOI:** 10.1007/s00018-026-06224-y

**Published:** 2026-05-09

**Authors:** Ruhao Yang, Niandan Hu, Hairui Chen, Wenqiang Li, Ting Zheng

**Affiliations:** 1 Department of Emergency, Renmin Hospital of Wuhan University, Wuhan, China; 2 Department of Endocrinology, Zhongnan Hospital of Wuhan University, Wuhan, China

**Keywords:** Sepsis-induced acute kidney injury, GATM, Glucose metabolism, PDK4, Mitochondrial injury

## Abstract

**Background:**

This study aimed to identify a key target gene in proximal tubule cells (PTCs) of sepsis-induced acute kidney injury (S-AKI) and elucidate the underlying mechanisms.

**Methods:**

We screened and analyzed GEO datasets and identified a key gene, GATM, in S-AKI. An S-AKI mouse model was established via intraperitoneal injection of lipopolysaccharide (LPS), and HK-2 cells were used for in vitro experiments. The role of GATM was evaluated using adeno-associated virus (AAV)-mediated overexpression in mice and plasmid-mediated overexpression in HK-2 cells. To identify the downstream target genes of GATM, transcriptome sequencing was conducted. Pathological evaluation was performed using hematoxylin–eosin (HE) and periodic acid–Schiff (PAS) staining. Protein levels were determined by Western blotting (WB), immunohistochemistry (IHC), and immunofluorescence (IF) assays; Apoptosis was evaluated by TUNEL staining; Mitochondrial morphology and function were assessed by transmission electron microscopy (TEM), JC-1 and MitoSOX assays. Lactate concentration and cellular ATP levels were measured.

**Results:**

Through analysis of four datasets (GSE151658, GSE247727, GSE220812, and GSE139061), GATM was identified as a key gene in PTCs during S-AKI. GATM expression was downregulated in both S-AKI mice and LPS-stimulated HK-2 cells. In vivo, GATM overexpression improved renal function, alleviated tubular damage, decreased the expression of KIM-1, IL-6, Caspase-3, and 4-HNE, and reduced mitochondrial injury. In vitro, HK-2 cell viability was enhanced, TUNEL-positive cells were reduced, and damaged mitochondria were decreased. Transcriptome sequencing revealed that the PDK4-mediated glycolysis pathway was a downstream target of GATM. GATM overexpression downregulated PDK4 expression, reduced glycolytic enzyme levels (p-PDHA, HK2, LDHA, GLUT1) and lactate, and increased ATP production. However, PDK4 overexpression in HK-2 cells abolished the protective effects of GATM, enhanced glycolysis, increased lactate levels, and reduced ATP production.

**Conclusion:**

GATM plays a protective role in S-AKI by inhibiting PDK4-mediated aerobic glycolysis, enhancing ATP production, and restoring energy metabolism and mitochondrial function in PTCs.

## Introduction

Sepsis is a life-threatening syndrome caused by a dysregulated host response to infection, frequently resulting in multi-organ dysfunction involving the kidneys, liver, heart, and lungs. Despite advances in antimicrobial therapy and critical care, sepsis remains a major global health burden, affecting tens of millions of individuals annually and causing mortality rates approaching 20%, ranking among the leading causes of in-hospital death [1, 2]. Sepsis-induced acute kidney injury (S-AKI) represents one of the most common complications of sepsis, accounting for 45–70% of all acute kidney injury (AKI) cases, with mortality exceeding 40% in critically ill patients [3]. Elucidating the mechanisms underlying S-AKI and identifying effective therapeutic targets are therefore of critical clinical importance.

Current evidence indicates that S-AKI pathogenesis is multifactorial, encompassing systemic inflammation, microcirculatory disturbances, renin–angiotensin–aldosterone system dysregulation, complement activation, and metabolic reprogramming [4]. Among these, metabolic reprogramming has emerged as a central feature of S-AKI, reflecting abnormal cellular energy utilization. Proximal tubular cells (PTCs) are the kidney’s most mitochondria-rich and metabolically active cell type. Under physiological conditions, PTCs rely predominantly on fatty acid oxidation (FAO)-driven oxidative phosphorylation (OXPHOS) to meet their high energy demands. During sepsis, however, PTCs undergo a metabolic shift from FAO/OXPHOS to aerobic glycolysis, resembling the Warburg effect [5, 6]. While this shift transiently supports energy supply and cell survival, persistent reliance on glycolysis leads to inefficient ATP production, lactate accumulation, and mitochondrial dysfunction. Dysfunctional mitochondria, in turn, exacerbate metabolic derangements, creating a vicious cycle that accelerates S-AKI progression [7]. Restoring PTCs’ normal metabolic state and mitochondrial function is thus a promising strategy for mitigating S-AKI [8].

Glycine amidinotransferase (GATM), a rate-limiting enzyme in creatine biosynthesis [9], is localized to the mitochondrial intermembrane space of PTCs. Mutations in GATM have been associated with mitochondrial abnormalities and tubular dysfunction, as observed in Fanconi syndrome, where aberrant mitochondrial deposition leads to renal failure [10]. GATM has also been implicated in polycystic kidney disease and chronic kidney disease [11, 12]. However, its role in AKI, particularly S-AKI, remains poorly understood. We performed an integrated analysis of four S-AKI-related GEO datasets and identified GATM as a key differentially expressed gene during S-AKI.

Pyruvate dehydrogenase kinase 4 (PDK4) is a pivotal regulator of glucose metabolism that inhibits pyruvate dehydrogenase (PDH), thereby promoting aerobic glycolysis and limiting pyruvate entry into mitochondria for OXPHOS [13]. As a member of the PDK family, PDK4 is a potential therapeutic target for multiple metabolic disorders [14]. Its precise role in S-AKI, however, remains unclear. Our findings suggest that GATM may regulate PDK4, raising the possibility that GATM mitigates S-AKI by modulating PDK4-mediated glucose metabolism.

In this study, we employed GATM-overexpressing mouse and PTC models to investigate the biological function of GATM in S-AKI. We further elucidated the GATM–PDK4 axis as a potential regulatory mechanism in S-AKI pathogenesis. This work provides mechanistic insight into metabolic remodeling in AKI and identifies GATM as a prospective therapeutic target for sepsis-induced kidney injury.

### Materials and methods

### Single-cell RNA Sequencing (scRNA-seq) analysis

The single-cell transcriptomic dataset GSE151658 was retrieved from the GEO database, which profiled renal tissues from mice with lipopolysaccharide (LPS)-induced septic acute kidney injury at 4 h, 27 h, and 36 h after LPS administration, along with sham samples. The downloaded gene expression matrices were analyzed using the Seurat R package. Quality control was performed by excluding genes expressed in fewer than five cells, cells expressing fewer than 300 genes, and cells with mitochondrial UMI counts exceeding 50% of total UMIs. Cells were clustered, resulting in 28 clusters. Cell types were annotated based on marker genes identified by FindAllMarkers, cross-referenced with the CellMarker database and previously published studies, yielding 13 distinct cell types. Differentially expressed genes (DEGs) in PTCs were identified using the subset and FindMarkers functions in Seurat. Cell-cell interaction analysis was performed using the CellChat package to identify intercellular communication among cell types.

### Bulk RNA Sequencing (Bulk RNA-seq) analysis

The bulk RNA-seq datasets GSE139061, GSE220812, and GSE247727 were obtained from the GEO database. DEGs were subsequently identified with DESeq2. Genes with an absolute fold change greater than 1 and a p-value less than 0.05 were considered DEGs. Gene Ontology (GO) enrichment analyses were conducted using Metascape (http://metascape.org/).

### Identification of overlapping genes

Overlapping DEGs among all datasets were identified using the Draw Venn Diagram online tool. A Venn diagram was generated to visualize the overlapping genes. (https://bioinformatics.psb.ugent.be/webtools/Venn/)

### Construction of the protein–protein interaction (PPI) Network and identification of hub genes

The intersected DEGs were uploaded to the STRING database (https://string-db.org/). The interaction data were downloaded and then imported into Cytoscape software (version 3.6.1; https://cytoscape.org/) for PPI network visualization. Subsequently, the cytoHubba plugin in Cytoscape was employed to identify hub genes.

### Experimental animals

Male C57BL/6 mice (8 weeks old) were purchased from Hunan SJA Laboratory Animal Co., Ltd. (Hunan, China). All mice were maintained under specific pathogen-free (SPF) conditions at a constant temperature of 23 ± 2 °C. All experimental procedures were approved by the Animal Ethics Committee of Renmin Hospital of Wuhan University (Number:20230205D). The mice were randomly divided (*n* = 6 per group). The LPS model group received an intraperitoneal injection of LPS (L2630, Sigma) at a dose of 10 mg/kg. The control group received an equal volume of normal saline. After 24 h, the mice were anesthetized and sacrificed.

### AAV-Mediated overexpression of GATM in mice

Male C57BL/6 mice (4 weeks old) were acclimated under SPF conditions. The adeno-associated virus for GATM overexpression (AAV-GATM) and the control virus (AAV-NC) were purchased from General Biol Co., Ltd. (Anhui, China). Each mouse received the virus via tail vein injection at a dose of 5 × 10¹¹ vg (diluted to about 100µL) per mouse. Mice were randomly assigned to four groups (*n* = 5 per group): (1) Control + AAV-NC, (2) Control + AAV-GATM, (3) LPS + AAV-NC, and (4) LPS + AAV-GATM. Four weeks after AAV administration, mice received LPS (10 mg/kg) stimulation. Twenty-four hours later, all mice were anesthetized and sacrificed.

### Cell culture and treatment

Human proximal tubular epithelial cells (HK-2) were purchased from the American Type Culture Collection (ATCC, USA) and maintained at the Institute of Nephrology and Urology, Wuhan University for subsequent experiments. HK-2 cells were cultured in DMEM/F12 with 10% fetal bovine serum and placed in the incubator with 5% CO_2_ at 37 °C. Cells in the LPS group were exposed to 10 µg/mL LPS for 24 h. After the treatments, cells were harvested for downstream analyses.

### Transient overexpression in HK-2 cells

The pcDNA3.1 and the control plasmid were purchased from General Biol Co., Ltd. (Anhui, China). For plasmid transfection, log-phase HK-2 cells in good condition were transfected with the GATM or PDK4 overexpression recombinant plasmids using Lipofectamine 3000 (L3000, Thermo Fisher). LPS treatment (10 µg/mL) was applied 24 h after transfection, and cells were subsequently harvested for downstream analyses.

### Histology and Staining

Fresh kidney tissues were fixed in polyformaldehyde for at least 24 h. After dehydrated through a graded ethanol series and xylene substitutes, tissues were sectioned at ~ 4 μm using a microtome. Hematoxylin and Eosin (H&E) and Periodic Acid–Schiff (PAS) staining were performed according to standard methods. Cortical tubular injury was scored in six sections per group, with ten randomly selected 400× fields per section, using the following scale: 0 = no injury, 1 = 1–25%, 2 = 26–50%, 3 = 51–75%, 4 = 76–100%.

### Transmission electron microscopy (TEM)

Fresh kidney cortical tissues were rapidly harvested (within 1–3 min) and cut into approximately 1 mm³ tissue blocks. Tissue blocks were immediately placed in TEM fixative for further fixation. Samples were examined under a TEM (HT7800, Hitachi) for ultrastructural analysis.

### Immunofluorescence (IF) and immunohistochemistry (IHC)

Paraffin-embedded kidney sections were deparaffinized and rehydrated. Sections were blocked with 10% serum at 37 °C for 30 min. Sections were incubated with primary antibodies against GATM (12801-1-AP, Proteintech, China), 4-HNE (Ab46545, Abcam), IL-6 (BA4339, Boster, China), and Caspase-3 (19677-1-AP, Proteintech) diluted in 10% serum at 4 °C overnight. The next day, sections were incubated with 488-conjugated secondary antibodies (Thermo Fisher, USA) or HRP-conjugated secondary antibodies at approximately 37 °C for 45 min. Images were captured under a fluorescence microscope or light microscope, and the stained area was quantified using ImageJ software.

### Western blotting (WB)

Samples were lysed in RIPA buffer (Beyotime, China), followed by homogenization (for tissues) or scraping (for cells). Lysates were centrifuged at 12,000 rpm for 5 min at 4 °C to collect the supernatant as the total protein extract. Protein concentrations were determined using a BCA assay. Equal amounts of protein were denatured by boiling, separated by SDS-PAGE, and transferred onto PVDF membranes. Membranes were blocked with 5% non-fat milk, then incubated overnight at 4 °C with the following primary antibodies: GATM (12801-1-AP, Proteintech), KIM-1 (ab47635, Abcam), SOD2 (F0508, Selleck), PDK4 (12949-1-AP, Proteintech), p-PDHA1 (F1658, Selleck), LDHA (ES11979, ELK, China), HK2 (F0228, Selleck), GLUT1 (21829-1-AP, Proteintech), CPT1α (15184-1-AP, Proteintech), ACOX1 (A8091, ABclonal), CD36 (A5792, ABclonal) and β-actin (AC026, ABclonal). Band intensities were quantified using ImageJ software.

### Assessment of renal function

Blood samples were collected from mice and allowed to clot at room temperature for 30 min. Samples were then centrifuged at 1,000 rpm for 5 min, and the supernatant was collected. Serum creatinine levels were determined using the creatininase–sarcosine oxidase enzymatic method with an automatic biochemical analyzer.

### Cell viability assay (CCK-8)

HK-2 cells were plated in 96-well plate and cultured overnight. Each condition was tested in six replicates, with blank controls included. Peripheral wells were filled with 100 µL of sterile PBS to maintain humidity and prevent edge effects. Following treatment, 10 µL of CCK-8 reagent (Beyotime, China) was added to each well and incubated for 2 h at 37 °C. Absorbance was measured at 450 nm using a microplate reader.

### Terminal deoxynucleotidyl transferase dUTP Nick-End labeling (TUNEL assay)

After cells had adhered and received the designated treatments. Cells were fixed with 4% paraformaldehyde for 30 min. The TUNEL reaction mixture was prepared according to the manufacturer’s instructions (Beyotime, China; Roche, USA), and 50 µL was applied to each slide, and then incubated in the dark for 60 min. Fluorescence images were captured under a fluorescence microscope. For quantification, six slides per group were analyzed, and ten randomly selected fields at 400× magnification per slide were evaluated, with apoptosis expressed as the number of TUNEL-positive cells per field area.

### Mitochondrial membrane potential assay (JC-1 assay)

JC-1 dye (HY-K0601, MCE) was brought to room temperature and 10 µL of JC-1 (200 µM) was added to each well to achieve a final concentration of 2 µM, followed by gentle mixing. Cells were incubated at 37 °C for 15–20 min. Cells were then observed using a confocal laser scanning microscope (Olympus, Japan). Mitochondrial membrane potential was expressed as the ratio of red to green fluorescence.

### Mitochondrial superoxide detection (MitoSOX Assay)

Cells were incubated with the MitoSOX (T75342, Targetmol) working solution (approximately 1 mL per well) at 37 °C in the dark for 15–30 min. Fluorescence images were captured using a confocal laser scanning microscope (Olympus, Japan), and mitochondrial superoxide levels were assessed based on red fluorescence intensity. The median fluorescence intensity was used to estimate the average production of mitochondrial superoxide.

### RNA Extraction and quantitative real-time PCR (qRT-PCR)

Total RNA was extracted from HK-2 cells using TRIzol reagent (15596018, Invitrogen). Complementary DNA (cDNA) was synthesized from 1 µg of total RNA using a qPCR-specific reverse transcription kit (Takara, Japan).

For quantitative PCR, the SYBR Premix Ex Taq™ II (Takara, Japan) was combined with cDNA and gene-specific primers, and the final reaction volume was adjusted to 20 µL with RNase-free water. qRT-PCR analysis was carried out using the CFX96 Touch Real-Time PCR Detection System. The relative mRNA levels were determined by the 2^−ΔΔCt^ method, with β-actin serving as the reference gene. The primer sequences applied in this experiment are provided below:

**Table:** 

Gene	Forward (5′→3′)	Reverse (5′→3′)
GATM	ACGAATGGGACCCCTTAGAGG	CCTTACTGTCACTCCTTCCGTT
KIM-1	TGTCTGGACCAATGGAACCC	GGCAACAATATACGCCACTGT
PDK4	AACTGTGATGTGGTAGCAGTGG	GATGTGAATTGGTTGGTCTGG

### Tissue transcriptome sequencing

For transcriptome analysis, kidney tissues were collected from three groups: (1) Control group, (2) LPS-induced AKI model group, and (3) LPS + AAV-GATM treatment group, with three mice per group. Transcriptome sequencing was performed at Personalbio (Shanghai, China).

### Transcriptome data analysis

DEGs were identified using the DESeq2 package in R, with thresholds set at |log2 fold change| > 2 and *p* < 0.05. Gene set variation analysis (GSVA) was performed to assess pathway-level changes. KEGG gene sets for Mus musculus were downloaded from the MSigDB database and analyzed using the msigdbr and GSVA packages, with heatmaps visualized using the pheatmap package. Gene set enrichment analysis (GSEA) was conducted using the clusterProfiler package, and results were visualized as bubble plots or pathway enrichment plots using ggplot2 and enrichplot. To identify mitochondrial-related genes, mouse mitochondrial gene sets were obtained from the MitoCarta database and intersected with the DEGs from transcriptome sequencing, with overlapping genes displayed using Venn diagrams.

### Lactate assay

Cell culture supernatants were collected, and lactate concentrations were measured (Nanjing Jiancheng, China). Absorbance was measured at 546 nm, and a standard curve was generated based on the known lactate concentrations. The lactate concentration of each sample was then calculated by interpolating the corresponding absorbance value on the standard curve.

### ATP assay

Cells were lysed and performed ATP assay (HY-K0314, MCE). A standard curve was generated by plotting ATP concentration (nM) against relative luminescence units (RLU) measured using a multifunctional plate reader. Sample ATP concentrations were then calculated based on the standard curve.

### Statistical analysis

All data are presented as mean ± standard deviation (SD) and were analyzed using GraphPad Prism 8.0 or SPSS 22.0. Differences among multiple groups were evaluated using one-way analysis of variance (ANOVA) with Tukey’s post hoc test, while comparisons between two groups were performed using an unpaired Student’s t-test. *p* < 0.05 was considered statistically significant.

## Results

### scRNA-seq analysis uncovered the cellular characteristics of the kidneys in mice with S-AKI

**Fig. 1:**
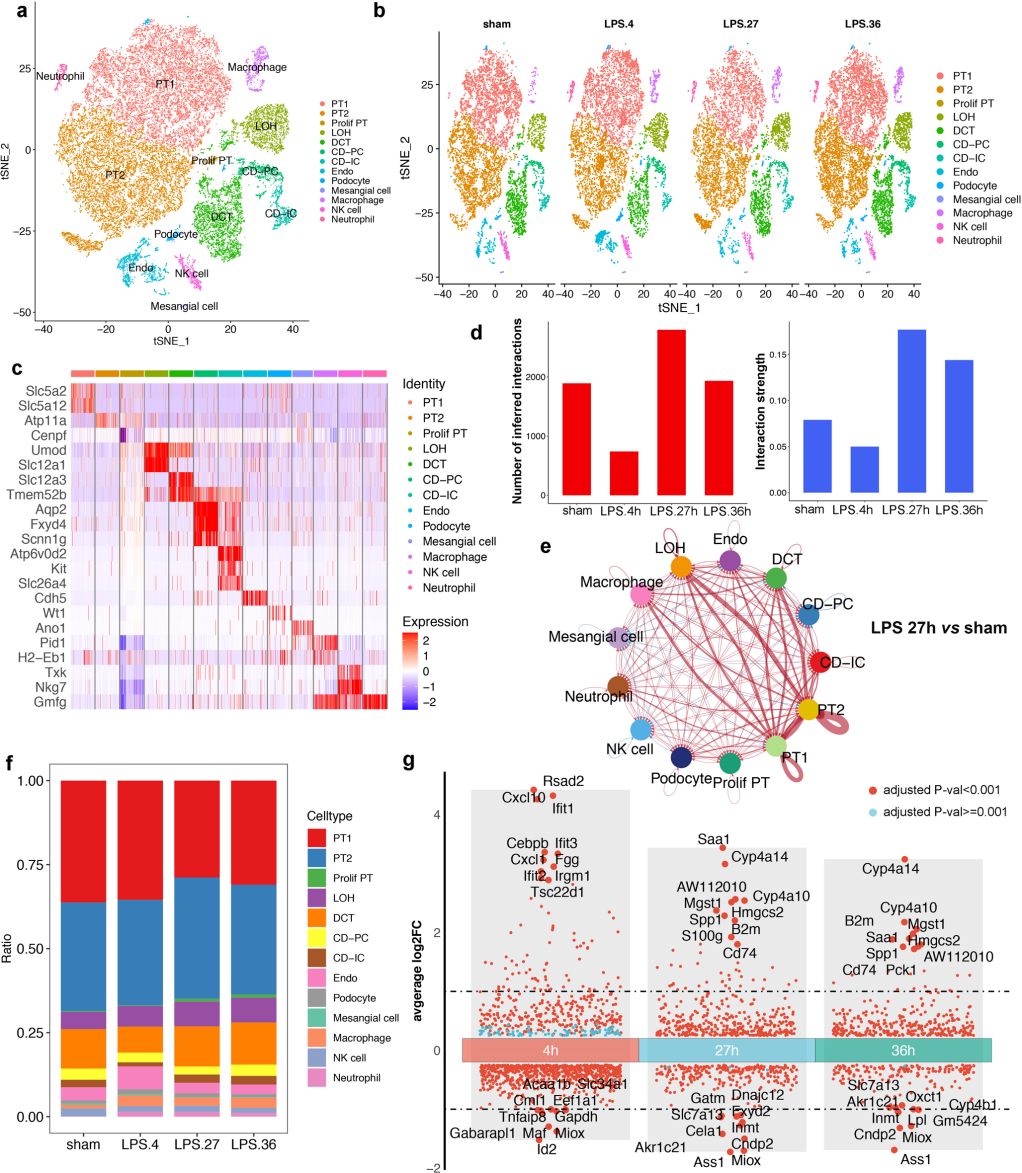
scRNA-seq analysis uncovered the cellular characteristics of the kidneys in mice with S-AKI. (**a**) The t-SNE plot displays the annotated cell types. S1/2 and S2/3 proximal tubules (PT1, PT2), proliferative PT (Prolif-PT), Loop of Henle (LOH), Distal convoluted tubules (DCT), Collecting duct principal cells (CD-PC), Collecting duct intercalated cells (CD-IC), Endothelial cells (Endo). (**b**) t-SNE plot from different groups. (**c**) The heatmap illustrates the marker genes corresponding to each cell type. (**d**) The bar plot shows the number and strength of cell–cell interactions in different groups. (**e**) The circular plot illustrates the cell-cell communication network in the LPS 27 h group compared to the control group, with red indicating upregulation and blue indicating downregulation of communication. (**f**) Distribution of cell type proportions in different groups. (**g**) The DEGs of PTCs in each group were identified, and the names of the top 10 upregulated and downregulated genes were annotated

ScRNA-seq data (GSE151658) were obtained from kidney tissues of S-AKI mice. Thirteen distinct cell clusters were identified (Fig. 1a-b). The heatmap showed the representative marker gene expression of each cell type (Fig. 1c). Next, we analyzed the number and strength of interactions among cell types in each group. The number and strength of interactions in the LPS 27 h group were the highest (Fig. 1d). We therefore analyzed cell–cell interactions in this group compared with the sham group using the "CellChat" R package. The results showed that interactions between PT1, PT2, and other cell types were significantly increased, suggesting that PTCs play a key role in S-AKI (Fig. 1e). Additionally, PTCs—including PT1, PT2, and Prolif-PT—accounted for the largest proportion of cell types in the kidney (Fig. 1f). Therefore, PTCs were further extracted for DEGs analysis. The top 10 up- and downregulated genes in PTCs after LPS treatment relative to the sham group are shown in Fig. 1g.

### Integrative analysis revealed that GATM is a key gene in S-AKI

**Fig. 2:**
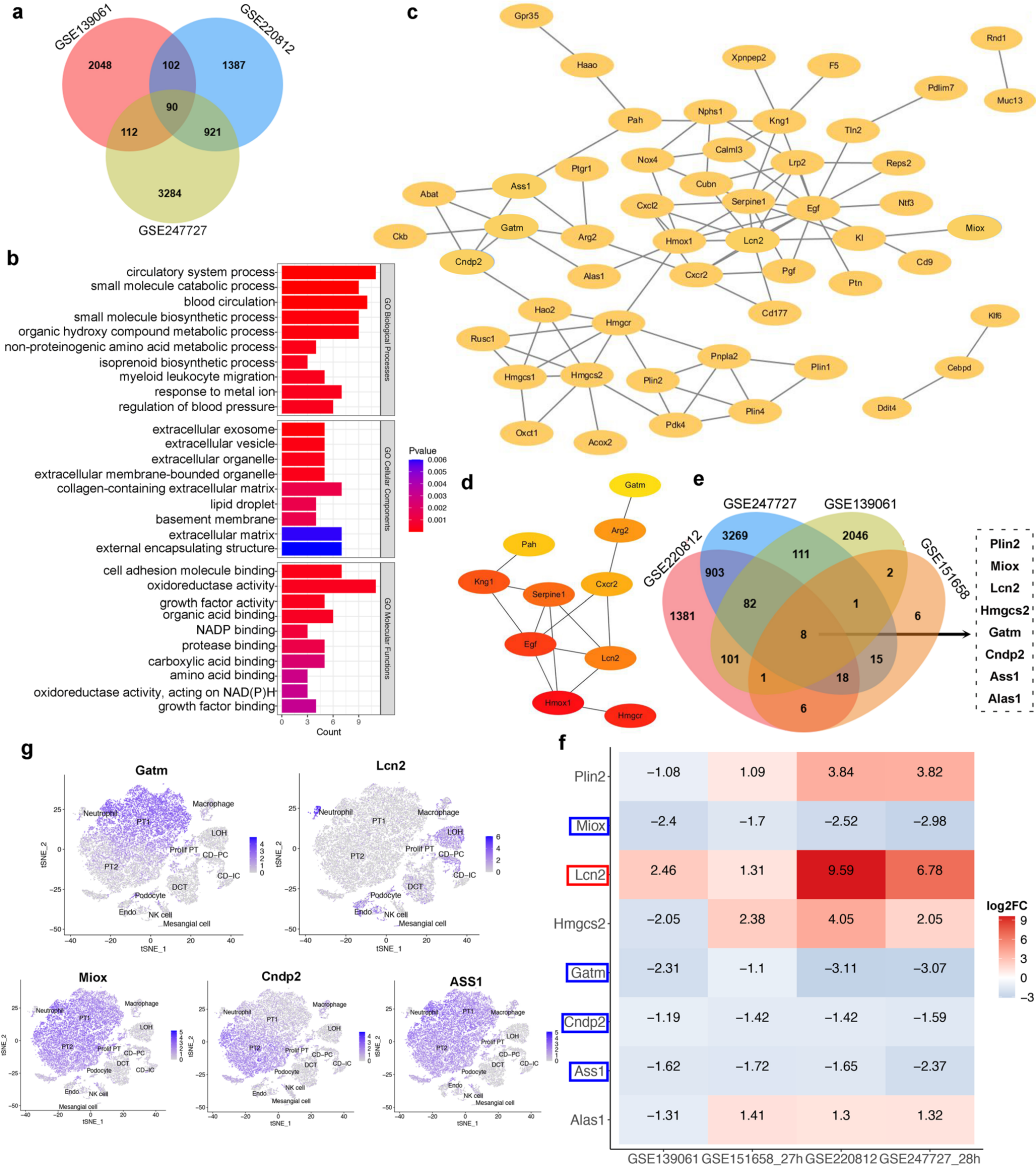
Integrative analysis revealed that GATM is a key gene in S-AKI. (a) The Venn diagram shows the common DEGs between the three bulk RNA datasets. (b) GO enrichment analysis of 90 common DEGs. (c) PPI network analysis of 90 common DEGs. (d) Top 10 hub genes identified from the PPI network. (e) The Venn diagram shows the DEGs common to a scRNA-seq dataset and three bulk RNA-seq datasets. (f) The heatmap shows the fold change in differential expression of the 8 intersecting genes identified in panel E across four datasets, with blue boxes for genes downregulated and red boxes for genes upregulated in all four datasets. (g) The t-SNE plot displays the expression distribution of the intersecting genes Gatm, Lcn2, Miox, Cndp2, and Ass1 across different cell types

Three bulk RNA-seq datasets from kidney tissues of S-AKI patients or mice were obtained, and their detailed information is shown in Table 1. Venn analysis identified 90 overlapping DEGs among the three datasets (Fig. 2a). Based on these 90 DEGs, GO enrichment analysis revealed the top 10 pathways across three GO categories (Fig. 2b). A PPI network constructed via STRING further identified ten hub genes, including LCN2, GATM, SERPINE1, EGF, HMOX1, HMGCR, KNG1, CXCR2, ARG2, and PAH (Fig. 2c–d).

**Table 1:** Datasets included in this study

Dataset	species	Model	Sequencing type
GSE151658	mouse	LPS	scRNA-seq
GSE247727	mouse	LPS	bulk RNA-seq
GSE220812	mouse	CLP	bulk RNA-seq
GSE139061	human	Sepsis-AKI	bulk RNA-seq

To identify potential target genes in PTCs, DEGs from PTCs in the scRNA-seq dataset were intersected with those from the three bulk RNA-seq datasets. This analysis identified eight overlapping genes, among which GATM and LCN2 were also included in the ten hub genes identified from bulk RNA-seq analysis (Fig. 2e). The fold changes of these eight intersecting genes are presented in Fig. 2f, and five of them, including GATM and LCN2, exhibited consistent expression patterns. The expression distribution of these five genes was further visualized using t-SNE plots (Fig. 2g). Given that LCN2 has been well characterized in AKI, whereas GATM remains poorly studied, we selected GATM for further investigation as a potential therapeutic target.

### The expression of GATM was downregulated in the S-AKI mouse model

**Fig. 3:**
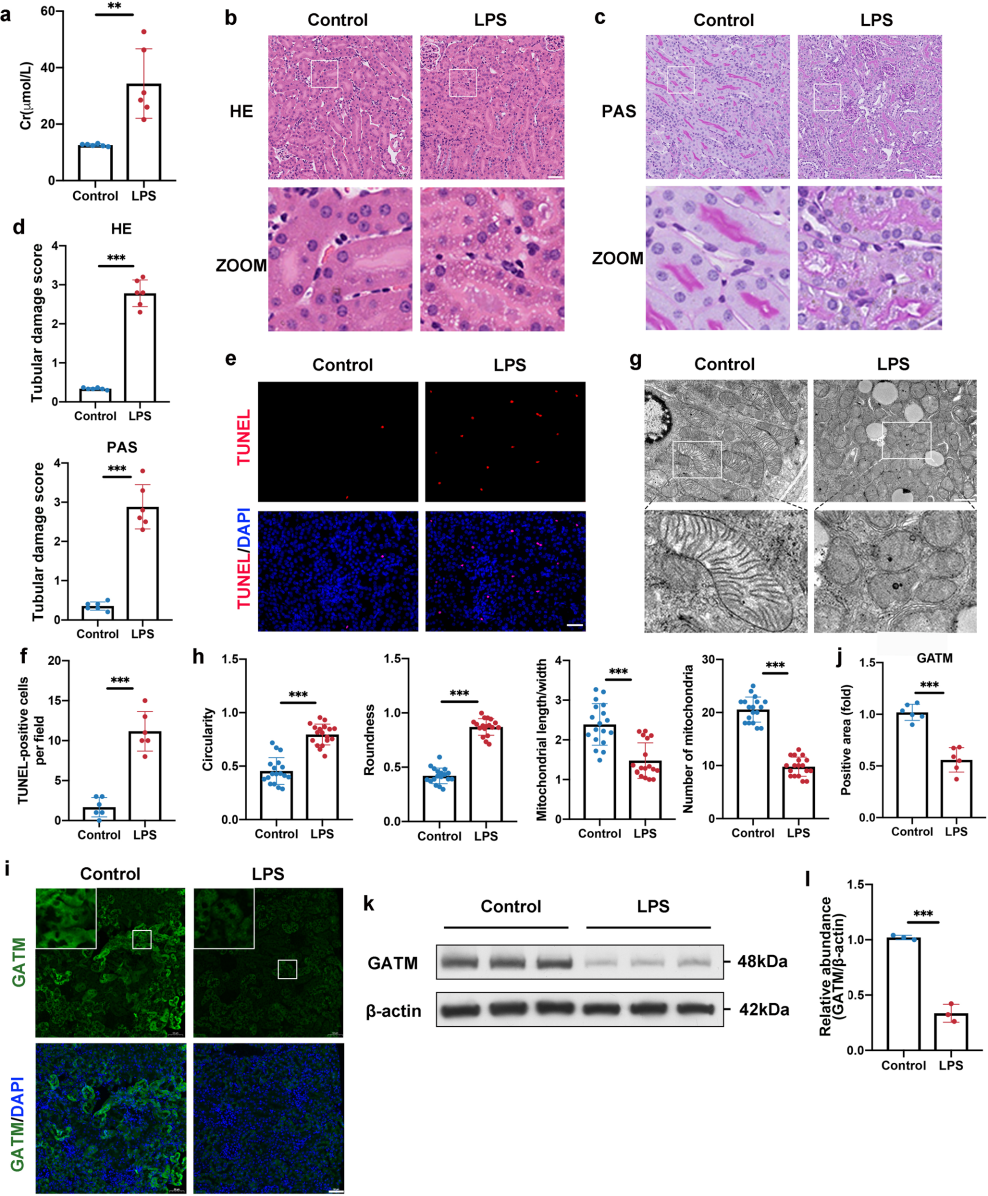
The expression of GATM was downregulated in the S-AKI mouse model. (**a**) Serum creatinine levels of mice in each group. (**b**) Representative microscopy images of H&E staining from each group. Scale bar = 50 μm. (**c**) Representative microscopy images of PAS staining from each group. Scale bar = 50 μm. (**d**) Quantitative analysis of tubular injury using H&E and PAS staining. (e) TUNEL staining from each group of mice, with blue fluorescence labeling the nuclei and red fluorescence labeling apoptotic cells. Scale bar = 50 μm. (**f**) Quantitative analysis of the number of TUNEL-positive cells in each group. (g) Representative TEM images showing the ultrastructure of mitochondria. Scale bar = 1 μm. (**h**) Mitochondrial circularity, roundness, aspect ratio, and number were quantified from TEM images of three random fields per mouse in each group. (i) Representative IF image of GATM protein, with green fluorescence labeling GATM and blue fluorescence labeling the nuclei. Scale bar = 50 μm. (**j**) Quantitative analysis of the relative expression levels of GATM based on panel i. (**k**) Representative WB bands of GATM protein. (**l**) The intensities of WB bands for GATM were quantified based on panel k. Data are presented as mean ± SEM, *n* = 6. ns, not significant; *p < 0.05; **p < 0.01; ***p < 0.001

Compared with the sham group, LPS-treated mice showed significantly elevated serum creatinine levels (Fig. 3a). Histological analyses revealed marked tubular epithelial cell (TEC) injury: H&E staining showed edema, vacuolar degeneration, blurred cell boundaries, and focal hemorrhage and congestion (Fig. 3b); PAS staining revealed loss of TEC brush border, tubular lumen dilation with protein casts (Fig. 3c). Correspondingly, tubular injury scores were significantly increased (Fig. 3d). TUNEL assay demonstrated a marked increase in apoptotic cells in LPS-treated kidneys (Fig. 3e–f). TEM analysis showed mitochondria with cristae loss, swelling, partial dissolution, and reduced aspect ratio, indicating fragmentation (Fig. 3g–h). IF and WB analyses further revealed a significant decrease in GATM expression in the LPS group (Fig. 3i–l).

### GATM overexpression alleviates S-AKI in a mouse model

**Fig. 4:**
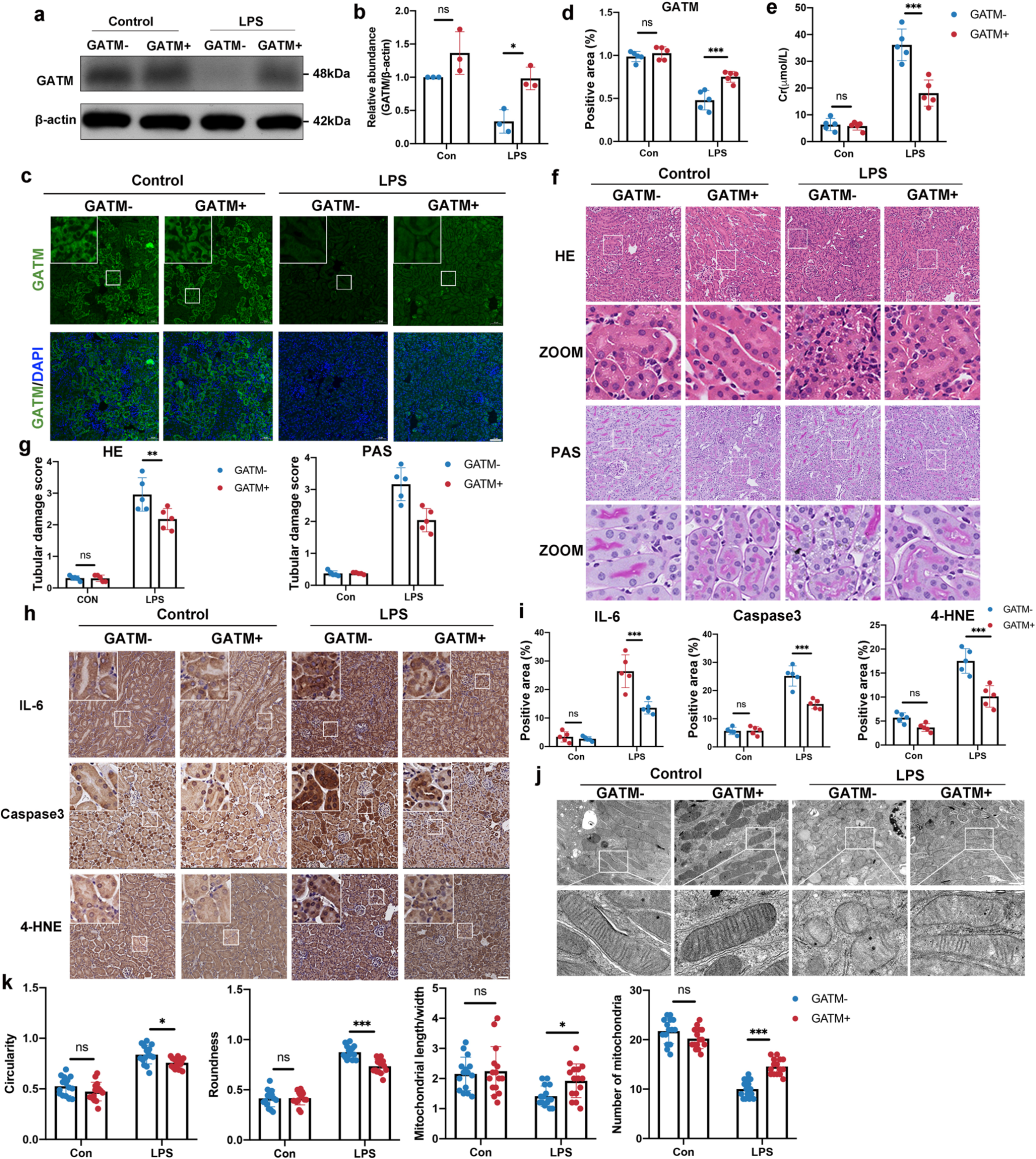
GATM overexpression alleviates S-AKI in a mouse model. (**a**) Representative WB bands of GATM protein. (**b**) The intensities of WB bands for GATM were quantified. (**c**) Representative IF image of GATM protein, with green fluorescence labeling GATM and blue fluorescence labeling the nuclei. Scale bar = 50 μm. (**d**) Quantitative analysis of the IF images based on panel c. (**e**) Serum creatinine levels of mice in each group. (**f**) Representative microscopy images of H&E and PAS staining from each group. Scale bar = 50 μm. (**g**) Quantitative analysis of tubular injury using H&E and PAS staining. (**h**) Representative IHC images of IL-6, caspase-3, and 4-HNE in each group. Scale bar = 50 μm. (**i**) Quantitative analysis of the relative expression levels of IL-6, caspase-3, and 4-HNE based on panel h. (**j**) Representative TEM images showing the ultrastructure of mitochondria. Scale bar = 1 μm. (**k**) Mitochondrial circularity, roundness, aspect ratio, and number were quantified from TEM images of three random fields per mouse in each group. Data are presented as mean ± SEM. *n* = 5. ns, not significant; *p < 0.05; **p < 0.01; ***p < 0.001

To investigate the role of GATM in S-AKI, we established a mouse model with GATM overexpression. AAV-GATM transduction significantly increased GATM expression (Fig. 4a–d). Compared with control group, LPS treatment increased serum creatinine, which was reduced by GATM overexpression (Fig. 4e). Histological analysis showed severe tubular injury in LPS-treated mice, which was alleviated by GATM overexpression (Fig. 4f–g). IHC staining revealed that LPS treatment significantly upregulated IL-6, 4-HNE, and Caspase-3, while these markers were downregulated in the LPS + AAV-GATM group, indicating reduced inflammation, apoptosis, and oxidative stress (Fig. 4h–i). TEM analysis showed aggravated mitochondrial damage in LPS-treated mice, characterized by cristae disruption, dissolution, and fragmentation, which was significantly alleviated by GATM overexpression (Fig. 4j–k).

### GATM overexpression alleviates HK-2 cell injury induced by LPS

**Fig. 5:**
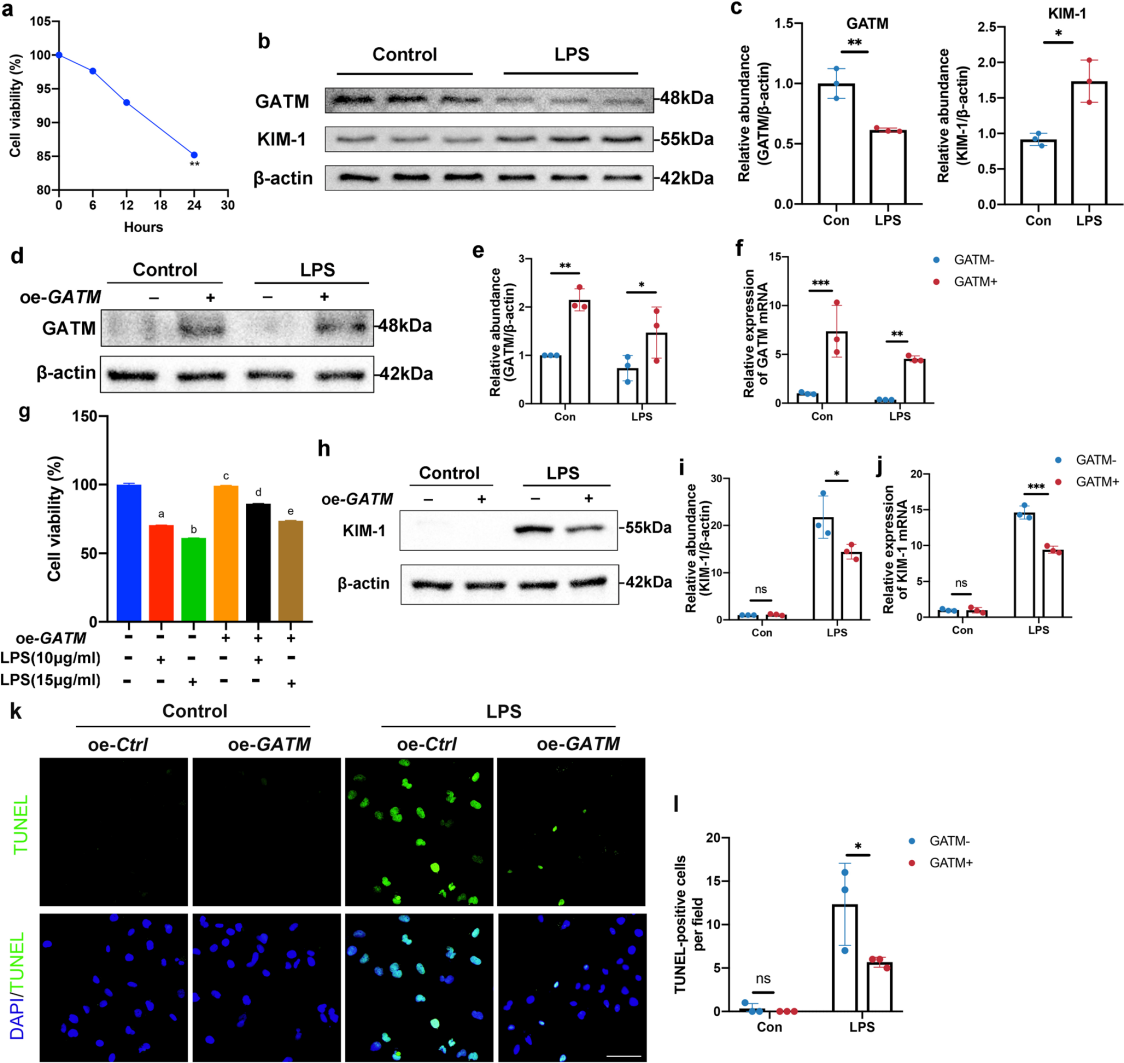
GATM overexpression alleviates HK-2 cell injury induced by LPS. (a) CCK-8 assay to detect cell viability at different time points. (b) WB analysis of GATM and KIM-1 protein expression levels in HK-2. (c) The intensities of WB bands based on panel b. (d) Representative WB bands showing GATM overexpression. (e) The intensities of WB bands based on panel d. (f) qPCR analysis of the relative expression of GATM mRNA in each group. (g) CCK-8 assay to assess cell viability under different LPS concentrations stimulation. (h) WB analysis of KIM-1 protein expression from each group. (i) The intensities of WB bands based on panel h. (j) qPCR analysis of the relative expression of KIM-1 mRNA in each group. (k) TUNEL staining to detect apoptosis, with green fluorescence labeling apoptotic cells and blue fluorescence labeling the nuclei. Scale bar = 100μm. (l) Quantitative analysis of the number of TUNEL-positive cells in each group. oe-Ctrl, overexpression control group; oe-GATM, overexpression GATM group. Data are presented as mean ± SEM, n=3. ns, not significant; *p < 0.05; **p < 0.01; ***p < 0.001; a: *p*<0.001, *vs* control; b: *p*<0.001, *vs* control; c: not significant, *vs* control; d: *p*<0.001, *vs* LPS (10 μg/ml); e: *p*<0.001, *vs* LPS (15 μg/ml)

In vivo, GATM overexpression alleviated renal injury. To verify this in proximal tubular cells, HK-2 cells were treated with LPS (10 µg/mL) for 6, 12, and 24 h, showing markedly reduced viability at 24 h (Fig. 5a). Thus, 10 µg/mL LPS for 24 h was used for subsequent experiments. Compared with the control, GATM expression decreased and KIM-1 increased in the LPS group, consistent with in vivo findings (Fig. 5b–c).

To evaluate GATM’s cytoprotective effect, HK-2 cells were transfected with a GATM overexpression plasmid, confirmed by qPCR and WB (Fig. 5d–f). After transfection, cells were stimulated with LPS (10 and 15 µg/mL). GATM overexpression significantly enhanced cell viability under both conditions (Fig. 5g). Additionally, GATM overexpression also reduced KIM-1 expression at both the mRNA and protein levels (Fig. 5h–j). TUNEL staining further showed fewer apoptotic cells in the LPS + OE-GATM group compared with the LPS group (Fig. 5k–l). These results confirm that GATM overexpression protects HK-2 cells during sepsis.

### Transcriptome analysis suggests that GATM may alleviate S-AKI by modulating metabolic pathways via PDK4

**Fig. 6:**
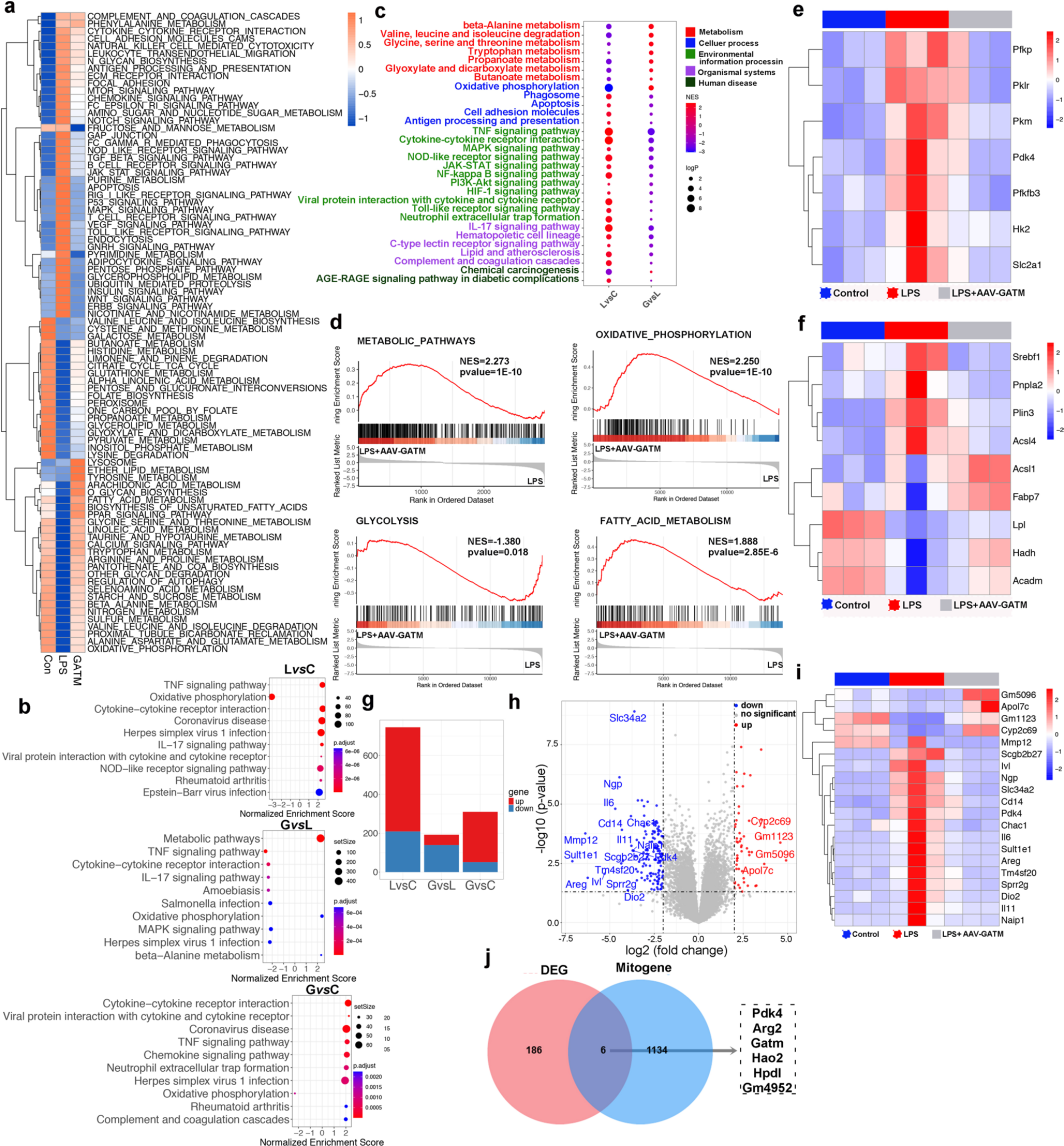
Transcriptome analysis suggests that GATM may alleviate S-AKI by modulating metabolic pathways via PDK4. (**a**) GSVA analysis of KEGG pathway activity in mouse kidneys. The color scale represents row Z-score–normalized GSVA enrichment scores (mean values for each group). (**b**) GSEA analysis shows the top 10 differential KEGG pathways among the three comparisons. (**c**) Changes in KEGG pathways across different comparisons. (**d**) GSEA plots display the enrichment scores for the "metabolic pathway", "oxidative phosphorylation pathway", "glycolysis" and "fatty acid metabolism". (**e**) The heatmap shows the expression of glycolysis-related genes in different groups. The color scale represents log2 fold change in gene expression. (**f**) The heatmap shows the expression of FAO-related genes in different groups. The color scale represents log2 fold change in gene expression. (**g**) Bar chart showing the number of DEGs across different comparisons. (**h**) Volcano plot showing DEGs between the LPS + AAV-GATM group and the LPS group, with the top 20 DEGs labeled. (**i**) Heatmap showing the expression of the top 20 DEGs in different groups based on panel h. Color indicates relative gene expression (log_2_(FPKM + 1), row-scaled). (**j**) Venn diagram showing the overlapping genes between DEGs (LPS + AAV-GATM group vs. LPS group) and mitochondria-related genes. Con, Control group; LPS, LPS group; GATM, LPS + AAV-GATM group; LvsC, LPS group vs. Control group; GvsL, LPS + AAV-GATM group vs. LPS group; GvsC, LPS + AAV-GATM group vs. Control group; NES, Normalized Enrichment Score

To explore how GATM alleviates S-AKI, transcriptome sequencing was performed on kidneys from control, LPS, and LPS + AAV-GATM mice. GSVA showed the KEGG pathway activity for the groups (Fig. 6a). GSEA analysis compared the top 10 differential KEGG pathways among three comparisons. Compared with controls, the LPS group showed activation of the TNF, IL-17, and cytokine–cytokine receptor interaction pathways, accompanied by suppression of oxidative phosphorylation. In contrast, GATM overexpression reversed these changes, enhancing metabolic and oxidative phosphorylation pathways while downregulating inflammatory signaling (Fig. 6b). Differential pathway alteration between LPS vs. control and LPS + AAV-GATM vs. LPS further supported these findings (Fig. 6c). Consistently, GSEA demonstrated that GATM overexpression in S-AKI mouse kidneys enhanced oxidative phosphorylation and fatty acid metabolism while suppressing glycolysis (Fig. 6d). The expression of glycolysis-related genes in the LPS group was upregulated, while it was suppressed under GATM overexpression (Fig. 6e). Most FAO-related genes showed the opposite trend (Fig. 6f). A total of 192 DEGs were identified in the LPS + AAV-GATM vs. LPS comparison (Fig. 6g). Among the top 20 DEGs, PDK4, CHAC1, and APOL7c were closely linked to metabolic regulation (Fig. 6h–i). Given that PDK4 plays a central role in glucose and fatty acid metabolism and is mitochondria-related (Fig. 6j), it was identified as a potential downstream target of GATM.

### Overexpression of GATM downregulates PDK4, inhibits aerobic glycolysis, and prevents lipid accumulation in the S-AKI mouse model

**Fig. 7:**
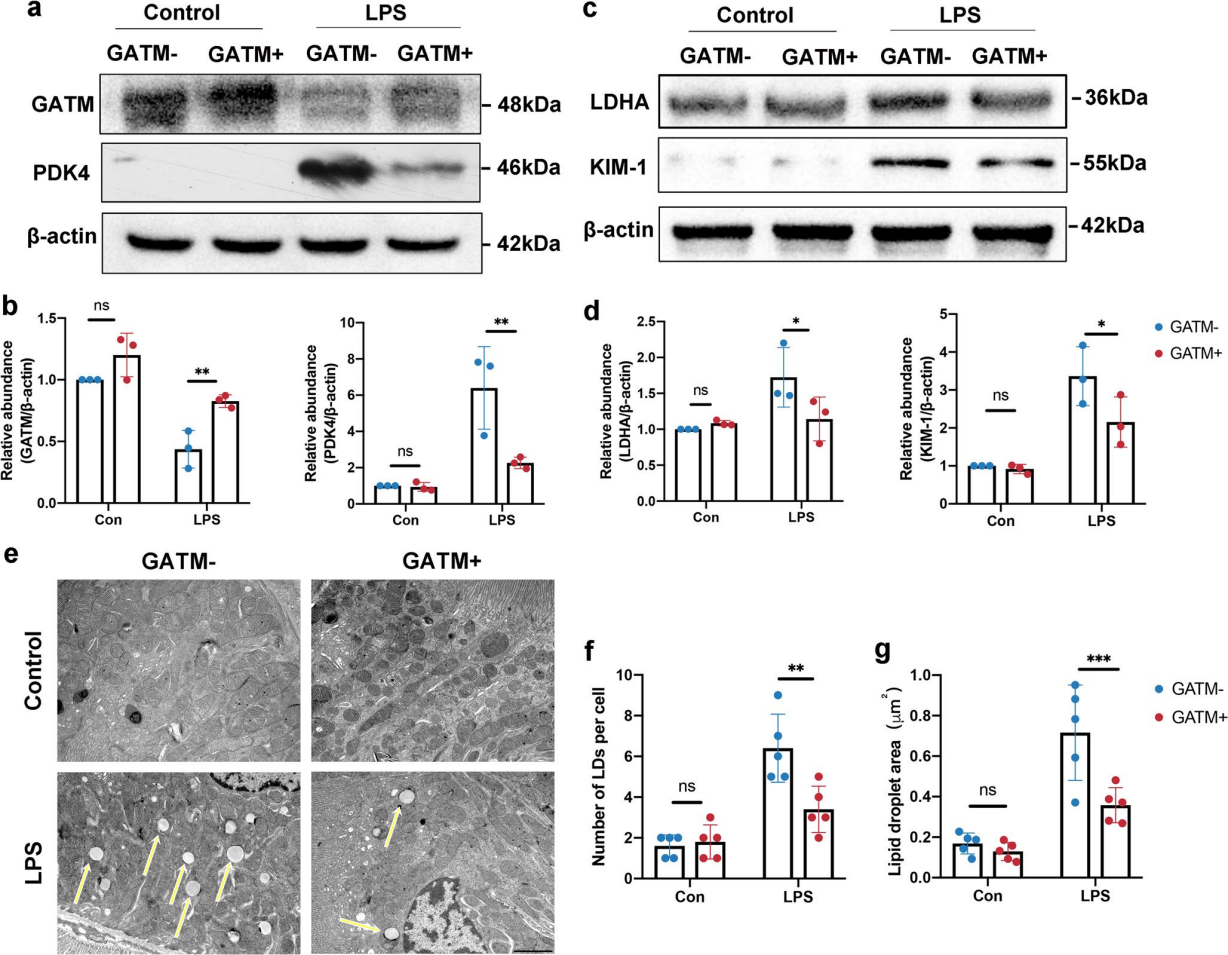
Overexpression of GATM downregulates PDK4, inhibits aerobic glycolysis, and prevents lipid accumulation in the S-AKI mouse model. (**a**) WB analysis of GATM and PDK4 protein expression levels in mouse kidneys. (**b**) The intensities of WB bands based on panel a. (**c**) WB analysis of LDHA and KIM-1 protein expression levels in mouse kidneys. (**d**) The intensities of WB bands based on panel c. (**e**) Representative TEM images showing the ultrastructure of lipid droplets, which are pointed out by yellow arrows, Scale bar = 2 μm. (**f**) Quantification of lipid droplet number per cell in different groups. (**g**) Quantification of lipid droplet area from three random fields per mouse in different groups. Data are presented as mean ± SEM, *n* = 5. ns, not significant; *p < 0.05; **p < 0.01; ***p < 0.001

Transcriptome analysis showed that GATM overexpression in mice reduced PDK4 expression. WB confirmed that PDK4 levels were lower in control kidneys but elevated in the LPS model. In the LPS + GATM group, PDK4 expression was significantly suppressed (Fig. 7a-b). To assess glucose metabolism, we examined lactate dehydrogenase A (LDHA) levels, which were higher in the LPS group, whereas GATM overexpression significantly reduced them, suggesting that GATM mitigates aerobic glycolysis. Additionally, KIM-1 was upregulated in the LPS group but lowered by GATM, reflecting protection against tubular injury (Fig. 7c-d). TEM further showed that LPS increased both the number and size of lipid droplets, while GATM reduced them, indicating decreased lipid accumulation (Fig. 7e–g).

### GATM overexpression mitigates aerobic glycolysis and mitochondrial injury in HK-2 cells stimulated by LPS

**Fig. 8:**
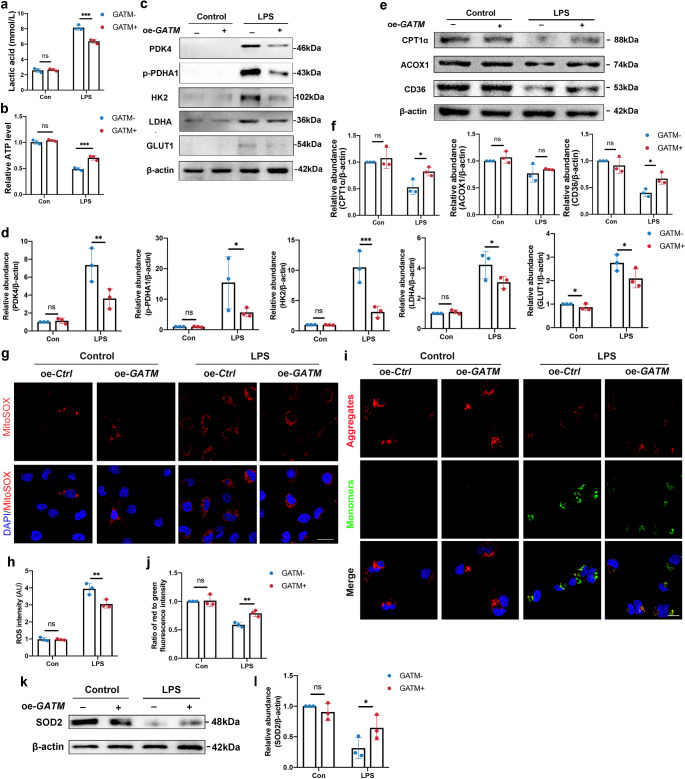
GATM overexpression mitigates aerobic glycolysis and mitochondrial injury in HK-2 cells stimulated by LPS. (**a**) Lactate levels in the medium of HK-2 cells. (**b**) ATP levels in the HK-2 cells of each group. (**c**) WB shows the protein levels of PDK4, p-PDHA, HK2, LDHA, GLUT1. (**d**) The intensities of the WB bands based on panel c. (**e**) WB shows the protein levels of CPT1α, ACOX1 and CD36. (**f**) The intensities of the WB bands shown in panel e. (g) MitoSOX staining displays oxidative stress levels in HK-2 cells. Red fluorescence indicates the accumulation of mitochondrial superoxide anions (reflecting oxidative stress levels), while blue fluorescence labels the nuclei. Scale bar = 50 μm. (**h**) Quantitative analysis of ROS intensity. (**i**) JC-1 staining displays MMP. Green fluorescence represents damaged mitochondria, while red fluorescence represents healthy mitochondria. Scale bar = 20 μm. (**j**) Quantitative analysis of MMP in each group. (**k**) WB showing the protein levels of SOD2. (**l**) The intensities of WB bands for SOD2 were quantified. oe-Ctrl, overexpression control group; oe-GATM, overexpression GATM group. Data are presented as mean ± SEM, *n* = 3. ns, not significant; *p < 0.05; **p < 0.01; ***p < 0.001

The renoprotective effect of GATM against S-AKI may involve modulation of the PDK4-mediated glycolytic pathway. In vitro, HK-2 cells were transfected with a GATM overexpression plasmid and exposed to LPS. Compared with control group, LPS stimulation markedly elevated extracellular lactate and reduced ATP production. Moreover, it increased PDK4 expression, PDHA phosphorylation (p-PDHA), and the levels of glycolytic enzymes (HK2, GLUT1, and LDHA), indicating impaired pyruvate flux into the tricarboxylic acid (TCA) cycle and a metabolic shift toward lactate accumulation. Conversely, GATM overexpression in LPS-treated cells markedly reversed these alterations by reducing PDK4 and glycolytic enzyme expression and lactate accumulation, while increasing ATP production. (Fig. 8a-d). GATM also affected lipid metabolism. LPS treatment significantly reduced key lipid enzymes (CPT1α, ACOX1, and CD36) compared with the control group. GATM overexpression in LPS-treated cells upregulated CPT1α and CD36, while ACOX1 levels showed no significant changes (Fig. 8e-f). These results suggest that GATM selectively enhances mitochondrial FAO and lipid uptake without affecting peroxisomal β-oxidation.

To assess mitochondrial injury, we evaluated oxidative stress and membrane potential. Compared with control group, MitoSOX staining revealed increased oxidative stress with LPS, which was attenuated by GATM (Fig. 8g-h). JC-1 staining revealed reduced mitochondrial membrane potential (MMP) in LPS-treated cells, as indicated by a lower red/green fluorescence ratio. GATM overexpression restored MMP (Fig. 8i-j). Superoxide dismutase 2 (SOD2), an antioxidant marker, decreased in LPS-treated cells but increased with GATM overexpression (Fig. 8k–l).

### PDK4 mediates the impact of GATM on glucose metabolism and abolishes its protective effect against LPS-induced damage

**Fig. 9:**
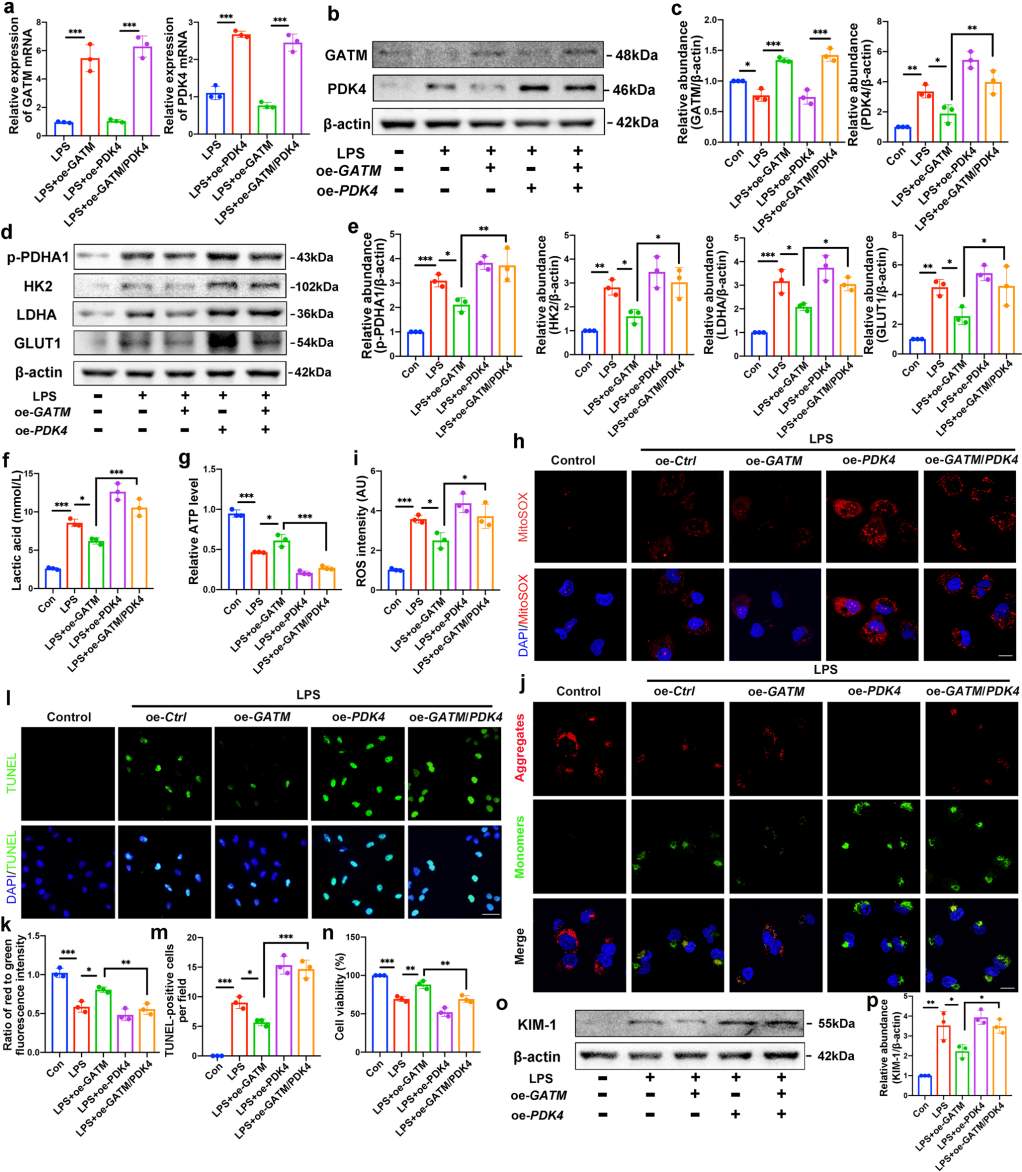
PDK4 mediates the impact of GATM on glucose metabolism and abolishes its protective effect against LPS-induced damage. (**a**) qPCR analysis of the relative expression of GATM and PDK4 mRNA in each group. (**b**) WB showing the protein levels of GATM and PDK4 in cells from each group. (**c**) The intensities of WB shown in panel b. (**d**) WB shows the protein levels of p-PDHA, HK2, LDHA, GLUT1. (**e**) The intensities of WB bands shown in panel d. (**f**) Lactate levels in the medium of HK-2 cells. (**g**) ATP levels in the HK-2 cells of each group. (**h**) MitoSOX staining showing oxidative stress levels in each group. Red fluorescence indicates the accumulation of mitochondrial superoxide anions (reflecting oxidative stress levels), while blue fluorescence labels the nuclei. Scale bar = 50 μm. (**i**) Quantitative analysis of ROS intensity. (**j**) JC-1 staining showing MMP. Green fluorescence represents damaged mitochondria, while red fluorescence represents healthy mitochondria. Scale bar = 20 μm. (**k**) Quantitative analysis of MMP in each group. (l) TUNEL staining displays apoptosis in HK-2 cells of each group, with green fluorescence labeling apoptotic cells and blue fluorescence labeling the nuclei. Scale bar = 50 μm. (**m**) Quantitative analysis of the number of TUNEL-positive cells in each group. (**n**) CCK-8 assay to assess cell viability. (**o**) WB shows the protein levels of KIM-1 in cells from each group. (**p**) The intensities of WB bands for KIM-1 were quantified. oe-Ctrl, overexpression control group; oe-GATM, overexpression GATM group; oe-PDK4, overexpression PDK4 group; oe-GATM/PDK4, GATM and PDK4 overexpression. Data are presented as mean ± SEM, *n* = 3. ns, not significant; *p < 0.05; **p < 0.01; ***p < 0.001

We previously showed that GATM downregulates PDK4, inhibits aerobic glycolysis, and enhances ATP production. Since PDK4 is a key glucose metabolism regulator, we hypothesized that GATM modulates glucose metabolism via PDK4 to alleviate S-AKI. To test this, HK-2 cells were transfected with GATM or PDK4 overexpression plasmids, or both, and exposed to LPS to assess glucose metabolism, mitochondrial injury, and GATM’s protective effects. Transfection efficiency was confirmed by qPCR and WB (Fig. 9a-c). Compared with the LPS group, the LPS + OE-GATM group showed reduced lactate, increased ATP, and lower expression of p-PDHA, HK2, LDHA, and GLUT1. Co-transfection with PDK4 reversed these effects, elevating lactate and glucose metabolism proteins while reducing ATP, indicating that PDK4 overexpression counteracts GATM’s metabolic regulation (Fig. 9d–g).

Next, we used MitoSOX and JC-1 staining to assess mitochondrial injury. Compared with the LPS group, MitoSOX and JC-1 staining showed that GATM decreased oxidative stress and restored MMP, whereas PDK4 co-expression abolished these protective effects (Fig. 9h–k). Furthermore, we measured apoptosis, cell viability, and KIM-1 expression to assess HK-2 cell injury. The LPS + OE-GATM group showed reduced apoptosis, increased cell viability, and lower KIM-1 expression. However, in the LPS + OE-GATM/PDK4 co-transfection group, GATM’s protective effects were significantly reduced (Fig. 9l-p).

## Discussion

Sepsis is a severe systemic syndrome caused by an uncontrolled host response to infection, frequently accompanied by AKI, and is characterized by high morbidity and mortality rates [1]. Currently, the treatment of S-AKI remains a major global clinical challenge because its pathogenesis is highly complex and not yet fully elucidated. Therefore, identifying potential therapeutic targets and clarifying their molecular mechanisms are of great significance for the prevention and treatment of S-AKI.

Among the nephron segments, PTCs play a pivotal role in the pathogenesis of S-AKI. PTCs are rich in mitochondria and require a substantial amount of energy to drive ion pumps that maintain water and electrolyte balance. Consequently, PTCs are vulnerable to ischemic and toxic insults [15, 16]. Moreover, histopathological studies have shown that in the early stages of sepsis, PTCs exhibit brush border loss, cytoplasmic vacuolization, and mitochondrial swelling [17], suggesting that PTCs are critical target cells during the progression of S-AKI. Based on these findings, we integrated scRNA-seq and bulk RNA-seq analyses to identify differentially expressed genes in PTCs during S-AKI and ultimately identified LCN2 and GATM as potential key regulatory genes. LCN2 encodes neutrophil gelatinase–associated lipocalin (NGAL) [18, 19]. Clinically, NGAL is widely used as a sensitive and early biomarker for AKI [20, 21]. However, the role of GATM in AKI remains unexplored. Thus, our study focused on elucidating the functional role of GATM in S-AKI. The GATM gene encodes L-arginine: glycine amidinotransferase, the rate-limiting enzyme in the first step of creatine biosynthesis. GATM is predominantly expressed in renal PTCs, with lower expression levels observed in the pancreas, liver, and central nervous system. Mutations in GATM are responsible for a rare hereditary disorder known as creatine deficiency syndrome (CDS) [9, 22]. Recent studies have shown that GATM participates in cancer [23–25], cardiovascular and cerebrovascular diseases [26, 27], obesity [28, 29], and statin-induced myopathy [30–32]. Increasing evidence also suggests a key role of GATM in kidney diseases [33]. Patients harboring heterozygous missense mutations in GATM often develop Fanconi syndrome accompanied by progressive renal failure. These mutations cause abnormal aggregation of GATM protein within the mitochondria of proximal tubular cells, forming pathological linear polymers and leading to renal dysfunction [10]. Additionally, downregulation of GATM exacerbates mitochondrial oxidative stress and cyst formation in polycystic kidney models [11], and its protein levels are positively correlated with estimated glomerular filtration rate (eGFR) in patients with chronic kidney disease (CKD) [12]. However, the role of GATM in AKI, particularly in S-AKI, has not yet been reported.

Our results demonstrated that GATM exerts a pronounced protective effect against S-AKI. However, the underlying mechanisms required further exploration. Transcriptomic analysis of kidney tissues revealed that GATM significantly altered cellular metabolic pathways by suppressing glycolysis and enhancing FAO and OXPHOS. These findings were further validated in both in vivo and in vitro experiments. Under normal physiological conditions, PTCs rely predominantly on FAO to meet their high energy demands for reabsorption of sodium, water, and glucose. During AKI, however, impaired FAO drives a metabolic shift toward enhanced aerobic glycolysis to compensate for the energy deficit [34]. In certain pathological states, despite adequate oxygen availability, pyruvate entry into the TCA cycle is reduced, leading to lactate accumulation—a phenomenon known as “aerobic glycolysis” [5, 35]. Upregulation of glycolytic enzymes and decreased TCA cycle metabolites have been observed in renal tubular cells during AKI resulting from sepsis and ischemia–reperfusion injury [36, 37]. In the early phase of sepsis, the metabolic switch toward aerobic glycolysis enhances infection tolerance and supports cell survival. However, prolonged reliance on this pathway results in detrimental effects, including lipid accumulation, tubulointerstitial fibrosis, and glomerulosclerosis [38, 39], ultimately impeding tubular regeneration and accelerating CKD progression. Therefore, inhibition of aerobic glycolysis and restoration of FAO- and OXPHOS-dominant metabolism represent effective therapeutic strategies for mitigating S-AKI. Previous studies have shown that suppressing glycolysis and promoting OXPHOS and FAO alleviate AKI [40], and delay AKI-to-CKD transition [41, 42].

To further elucidate the mechanism by which GATM regulates cellular energy metabolism, we performed transcriptome profiling and identified PDK4 as a critical downstream target of GATM. Both in vivo and in vitro validation confirmed that PDK4 expression was significantly downregulated upon GATM overexpression. PDK4 is localized in the mitochondrial matrix and inhibits PDH activity through phosphorylation, thereby reducing pyruvate flux into the TCA cycle and promoting aerobic glycolysis. Numerous studies have demonstrated that PDK4 plays a vital role in cellular energy metabolism. For example, PDK4 upregulation in tumor cells promotes aerobic glycolysis, enhances proliferation, and confers anti-apoptotic properties [43]. In senescent cells, PDK4 activation leads to lactate accumulation and has been identified as a potential target for delaying cellular aging [13]. In renal pathophysiology, PDK4 is also implicated in disease progression. In diabetic kidney disease, PDK4 promotes disease advancement by depleting the antioxidant regulator Nrf2 [44]. During ischemia–reperfusion injury, PDK4 facilitates succinate accumulation, resulting in mitochondrial dysfunction and excessive reactive oxygen species (ROS) production [45]. In cisplatin-induced AKI, PDK4 mediates mitochondrial oxidative stress and aggravates nephrotoxicity [46]. However, the role of PDK4 in S-AKI has not been previously reported. To verify whether GATM alleviates S-AKI through PDK4-mediated metabolic regulation, we overexpressed PDK4 in HK-2 cells. The results demonstrated that PDK4 overexpression promoted aerobic glycolysis, exacerbated mitochondrial injury, and increased PTC damage and apoptosis, ultimately abolishing the protective effects of GATM in S-AKI.

Nevertheless, some limitations of this study should be acknowledged. First, we primarily relied on gain-of-function approaches to investigate the role of GATM in S-AKI. Although these experiments demonstrate that restoration of GATM is sufficient to ameliorate S-AKI, loss-of-function strategies will be required in future studies to further clarify the endogenous contribution of GATM to S-AKI pathogenesis. Second, although our rescue experiments establish a functional regulatory axis in which PDK4 acts downstream of GATM in S-AKI, the mechanism by which GATM regulates PDK4 remains unclear. Future studies are necessary to investigate how GATM regulates PDK4.

## Conclusion

**Fig. 10:**
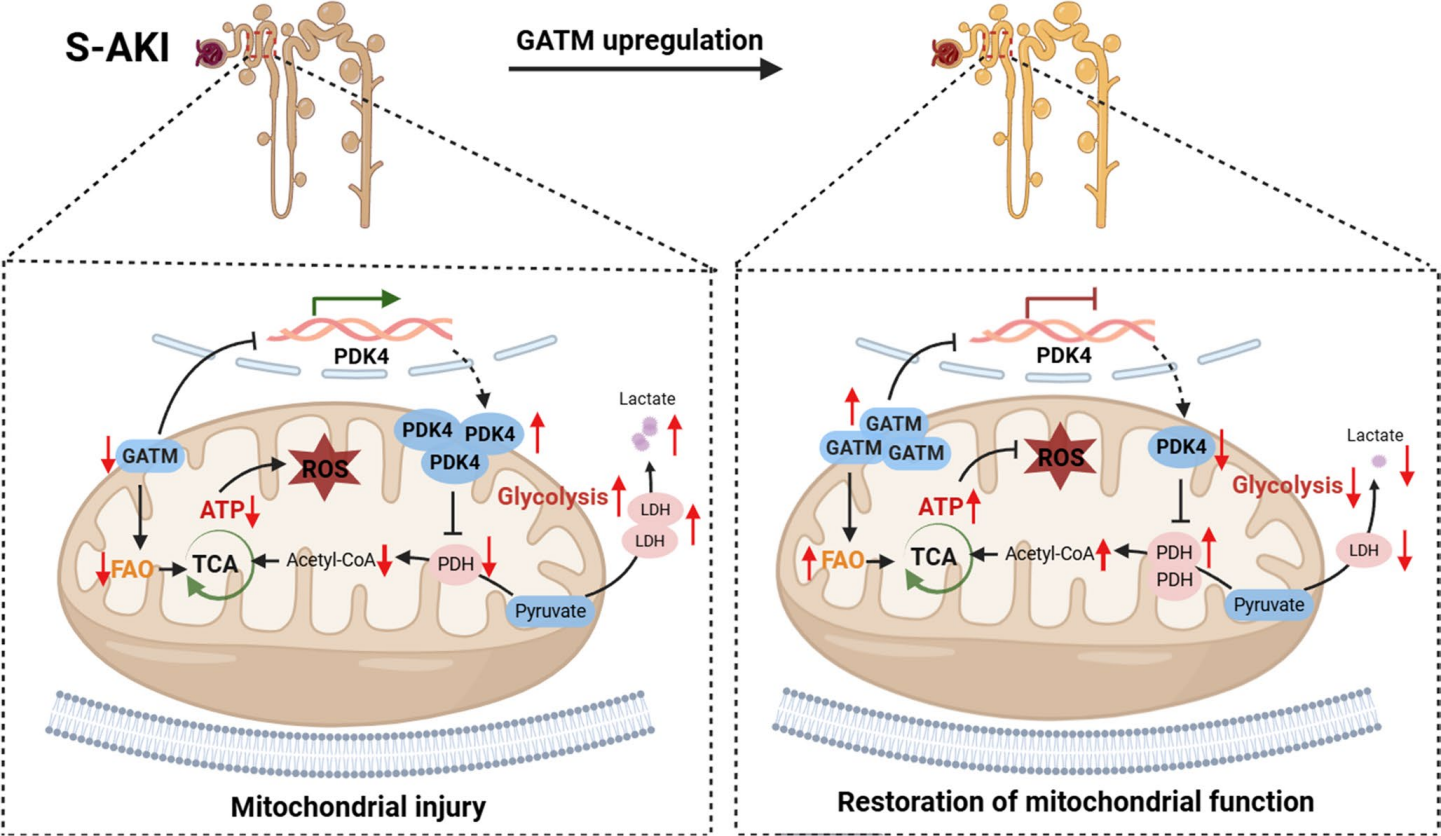
Mechanistic illustration of how GATM regulates glucose metabolism via PDK4 to alleviate S-AKI In S-AKI, downregulation of GATM promotes the transcription of PDK4, resulting in increased PDK4 expression. PDK4 inhibits PDH activity, thereby obstructing the entry of pyruvate into the TCA cycle and reducing ATP production. Concurrently, aerobic glycolysis is enhanced, leading to excessive conversion of pyruvate to lactate and subsequent lactate accumulation. In addition, fatty acid oxidation (FAO) is inhibited. These changes lead to elevated mitochondrial oxidative stress. Overexpression of GATM reverses these changes by suppressing PDK4 expression, restoring PDH activity, promoting pyruvate entry into the TCA cycle, enhancing mitochondrial ATP production, reducing lactate accumulation, and partially recovering FAO, thereby alleviating mitochondrial oxidative stress and restoring mitochondrial function in S-AKI. Red arrows indicate upregulation or downregulation of molecules. Black arrows indicate promotion. Horizontal bars with a vertical end indicate inhibition. Black dashed arrows indicate molecular translocation. FAO: fatty acid oxidation; TCA: tricarboxylic acid; ROS: reactive oxygen species; PDK4: pyruvate dehydrogenase kinase 4; PDH: pyruvate dehydrogenase; LDH: Lactate Dehydrogenase. (Created in https://BioRender.com)

Our study elucidates the protective mechanism of GATM in S-AKI and highlights the central regulatory role of the GATM–PDK4 signaling axis in metabolic reprogramming. GATM alleviates S-AKI by suppressing PDK4-mediated aerobic glycolysis, maintaining energy metabolic homeostasis, restoring mitochondrial function, and reducing PTC injury and apoptosis (Fig. 10). These findings provide new insights into the metabolic regulation of S-AKI and identify GATM as a promising therapeutic target.

## Data Availability

The RNA-seq datasets generated and analyzed during the current study are available in the ArrayExpress repository under accession number E-MTAB-16138. Requests for additional original data used in this study can be directed to the corresponding author.

## Abbreviations

S-AKI: Sepsis-induced acute kidney injury
AKI: Acute kidney injury
PTCs: Proximal tubular cells
FAO: Fatty acid oxidation
OXPHOS: Oxidative phosphorylation
GATM: Glycine amidinotransferase
PDK4: Pyruvate dehydrogenase kinase 4
PDH: Pyruvate dehydrogenase
scRNA-seq: Single-cell RNA sequencing
LPS: Lipopolysaccharide
DEGs: Differentially expressed genes
Bulk RNA-seq: Bulk RNA sequencing
PPI: Protein–protein interaction
HK-2: Human proximal tubular epithelial cells
TEM: Transmission electron microscopy
CCK-8: Cell viability assay
TUNEL assay: Terminal deoxynucleotidyl transferase dUTP nick-end labeling
MitoSOX assay: Mitochondrial superoxide detection
GSVA: Gene set variation analysis
GSEA: Gene set enrichment analysis
MMP: Mitochondrial membrane potential
SOD2: Superoxide dismutase 2
NGAL: Neutrophil gelatinase–associated lipocalin
CDS: Creatine deficiency syndrome
eGFR: Estimated glomerular filtration rate
CKD: Chronic kidney disease
TCA: Tricarboxylic acid
ROS: Reactive oxygen species
